# Supplementary material for: Cross-platform comparison of arbitrary quantum states
Source: Nat Commun. 2022 Nov 4;13:6620. doi: 10.1038/s41467-022-34279-5 (PMC9636372; doi:10.1038/s41467-022-34279-5)
Supplement: Supplementary file 1 — Supplementary Information [file 41467_2022_34279_MOESM1_ESM.pdf]

# Supplementary Information : Cross-Platform Comparison of Arbitrary Quantum States

## S1 The greedy method in the regime $M_S \gg 2^N$

The parameters $M_U$ and $M_S$ can be optimized through minimizing the statistical error with grid search [1,2] or using the importance sampling with partial information on the quantum state [3]. Both approaches require prior knowledge or simulation of the target state. Here, we devise a greedy method for sampling the unitary operation $U$ that reduces the statistical error without prior knowledge of the target state. The statistical error as a function of $M_U$ converges faster than uniformly sampling the unitary operation when the number of shots $M_S \gg 2^N$, where $N$ is the number of qubits. Therefore, the greedy method is particularly useful for the 5- and 7-qubit experiments. In this section, we demonstrate the comparison between the greedy method and random method for 5-qubit GHZ state.

When performing the fidelity estimation using randomized measurement, there are two major sources of errors, the shot noise error and the error from the incomplete tomography. The shot noise error can be suppressed when the number of shots is $M_S \gg 2^N$. Instead of uniformly sampling the random unitary from a set of unitary operators $U$, we generate a sequence of unitary operators while maximizing the distance between each random unitary. Specifically, we define the distance between two unitary operators as $d(u_a, u_b) = \max_\rho ||u_a \rho u_a^\dagger - u_b \rho u_b^\dagger||_1$. We sequentially generate the $M_U$ unitary operators $\{u_i\}$, where $1 \leq i \leq M_U$ sequentially. For $i = 1$, we sample a unitary

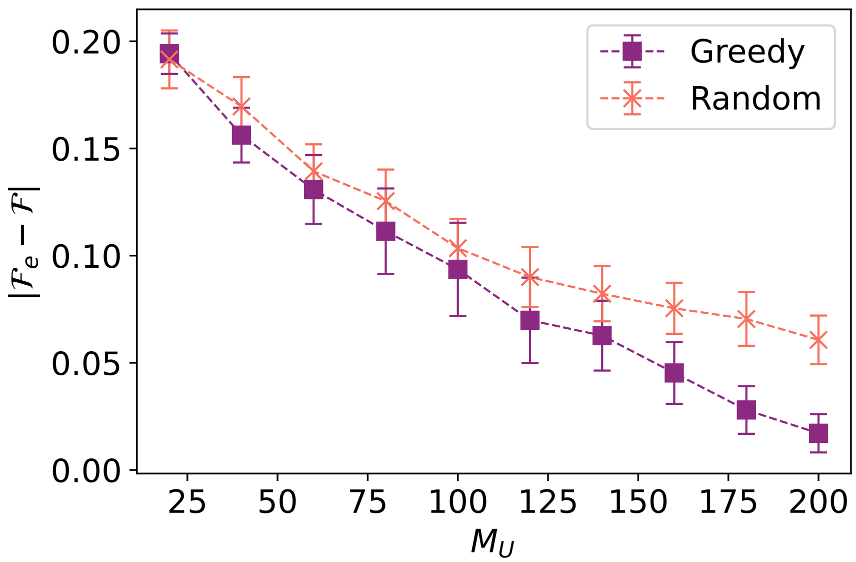


Figure S1: Comparison of error scaling for the fidelity of the GHZ states generated from UMD_1 vs IBM_1 with greedy or random sampling method for $M_U$.

operator randomly from $V$. For $i > 1$, we search for a unitary operator $u_i$ that minimizes the cost function $C(u_i; u_1, \ldots, u_{i-1}) = -\sum_{j=1}^{i-1} d(u_i, u_j)$. In order to minimize the cost function efficiently, we randomly generate $N_{\text{sample}}$ distinct unitary operators $u_{i,x}$, where $1 \leq x \leq N_{\text{sample}}$ and we define $u_i = \min_{u_{i,x}} C(u_{i,x}; u_1, \ldots, u_{i-1})$. In practice, we find that $N_{\text{sample}} = 200$ is enough to find the minimum for $N = 7$ and $V = Cl(2)^{\otimes N}$, where $Cl(2)$ is the single qubit Clifford group. The greedy method is summarized in Algorithm 1.

**Algorithm 1** Greedy method for sampling random unitary

**Input :** Number of random unitaries $M_U$, a set of unitary operators $S$

**Output :** $M_U$ random unitary operations for randomized measurement $\{u_i\}$, where $1 \le i \le M_U$.

1 : Sample $u_1$ randomly from $S$.

2 : **for** $i = 2$ **to** $M_U$ **do**

3 : Find a unitary $u_i \in S$ to minimize the cost function $C(u_i; u_1, \ldots, u_{i-1})$.

4 : **end for**

5 : **return** $\{u_i\}$

We compare the two different methods of sampling the random unitary $U$: the randomized sampling and the greedy method. Using these two methods, we evaluate the fidelity between the states prepared on the UMD_1 system and the IBM_1 system, by sampling subsets of various sizes $M_U$ from the full state tomography measurements. Figure S1 shows the error of the fidelity estimation between UMD_1 and IBM_1 as a function of $M_U$ for $M_S = 2000$. We see that the greedy method outperforms the random method in this regime.

To further characterize the performance of the greedy method, we perform numerical simulation through Pauli basis measurements. Specifically, to generate the random Pauli measurement using the greedy method, we define a set of unitary operators $S = \{H, HSH, I\}$ to perform measurement in the $x$, $y$, and $z$ basis. Using Algorithm. 1, we can sample the greedy random unitary operators.

We compare the measurement results for the greedy method and the random sampling method

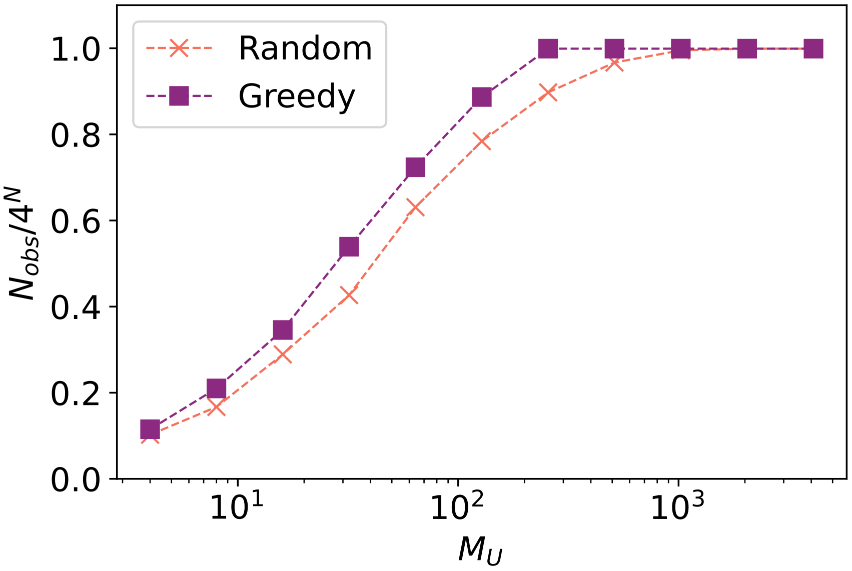


Figure S2: The number of Pauli-string operators that can be predicted through the set of measurements ($N_{obs}$) as function of $M_U$. In the large $M_U \gg 3^N$ limit, the measurement is equivalent to full state tomography and therefore, all the Pauli-string operator can be predicted ($N_{obs}/4^N = 1$). However, in the regime $M_U < 3^N$, we show that Greedy method can predict more observables than the random method.

in the regime $M_S \gg 2^N$. First, we compute the number of Pauli-string operators that can be predicted [2] through the set of measurements ($N_{obs}$). In Fig. S2, we present the $N_{obs}$ as function of $M_U$, normalized by the total number of Pauli string operators $4^N$. We see that the greedy method can predict more observables than the random method in the regime, $M_U < 3^N$. However, when $M_U \gg 3^N$, both greedy and random method can predict all $4^N$ Pauli string observables. Therefore, the shot noise is the dominant error source.

Second, we perform the simulation for the prediction of the linear observable $\mathrm{tr}(O\rho)$ and

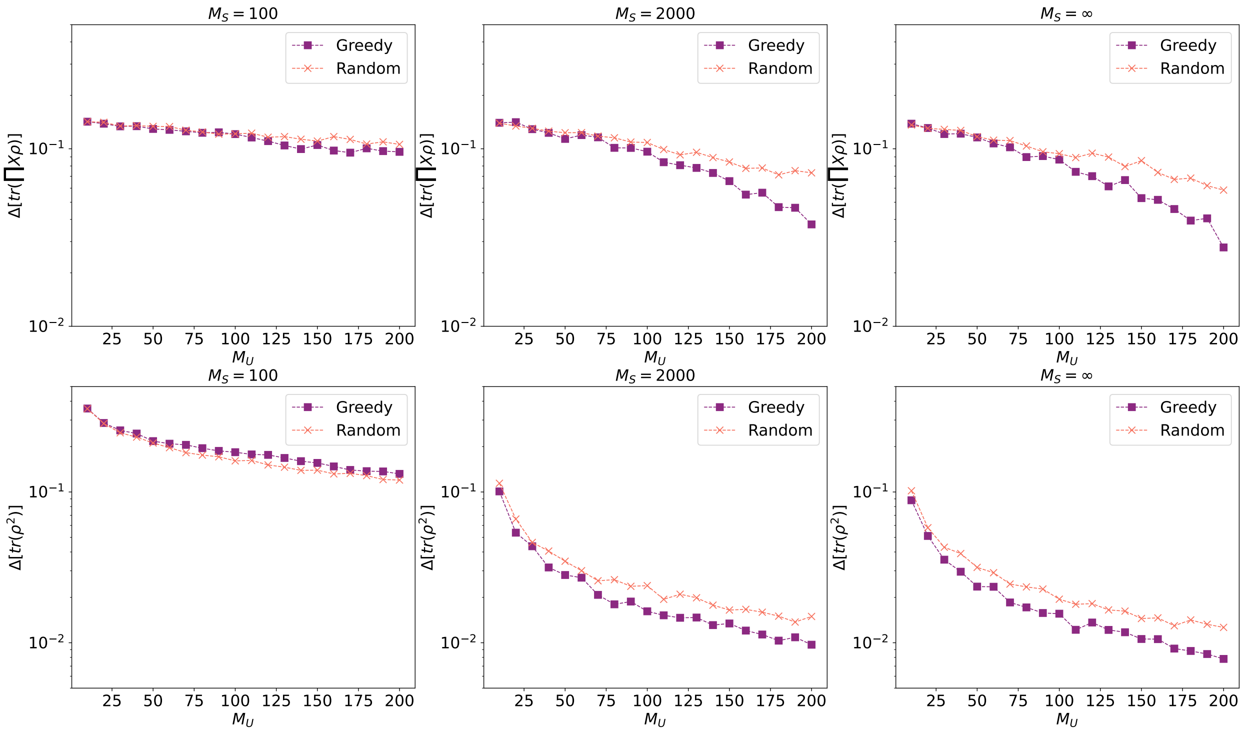


Figure S3: The measurement error $\Delta$ for linear observable $tr(O\rho)$, where $O = \bigotimes_{i=1}^{N} X_i$ and non-linear observable $tr(\rho^2)$. In the regime $M_S \sim O(2^N)$, the greedy and the random methods have similar performance. However, when the number of shots $M_S \gg O(2^N)$, the greedy method shows better performance against the randomized measurement method.

non-linear observable $\mathrm{tr}(\rho^2)$ [2], using both the greedy method and the random method. We first generate a random wave function $|\psi\rangle = U|0\rangle$, where $U$ is a unitary operator sampled from a Haar random distribution. We then generate $M_U$ unitary operators via both greedy and random methods and simulate the measurements implied by the unitary operators with $M_S$ shots. In Fig. S3, we present the average error $\Delta[O] = avg(|O_{measure} - O_{exact}|)$, where $O_{measure}$ is the expectation value of $O = \bigotimes_{i=1}^{N} X_i$ using randomized measurements and $O_{exact}$ is the exact expectation value. To obtain the average performance, we perform 100 independent numerical experiments and average

over the error from each numerical experiment.

## S2 Full state tomography vs. randomized measurement for 5-qubit GHZ state

Here, we compare the cross-platform fidelity obtained from full-state tomography and that from the randomized measurement on the 5-qubit GHZ state prepared on different platforms. We perform the full-state-tomography on a platform by measuring all of the 243 independent 5-qubit Pauli operators. To do so, we first independently generate the 5-qubit GHZ state circuits on each platform. Then we append different single-qubit rotations to the circuit to create the 243 different circuits. Each of the circuits gives the projective measurement result of one of the 243 independent 5-qubit Pauli operators. We set $M_S = 2000$ for all the platforms. For the randomized measurement, because a random Pauli basis measurement is equivalent to a randomized measurement with single qubit Clifford gates [2], we directly sample from the 243 Pauli basis measurements used for the full state tomography.

We calculate the cross-platform fidelity as a function of the number of randomized measurements $M_U$. The fidelity error $|\mathcal{F}_e - \mathcal{F}|$ is defined as the difference between the fidelity estimated by the randomized measurement $\mathcal{F}_e$ and the fidelity calculated through full state tomography $\mathcal{F}$. The average of $|\mathcal{F}_e - \mathcal{F}|$ and the standard deviation are calculated through a bootstrap resampling method [4]. The result (Fig. S4) shows that with only a fraction of the full state tomography measurements, one can estimate the cross-platform fidelity accurately.

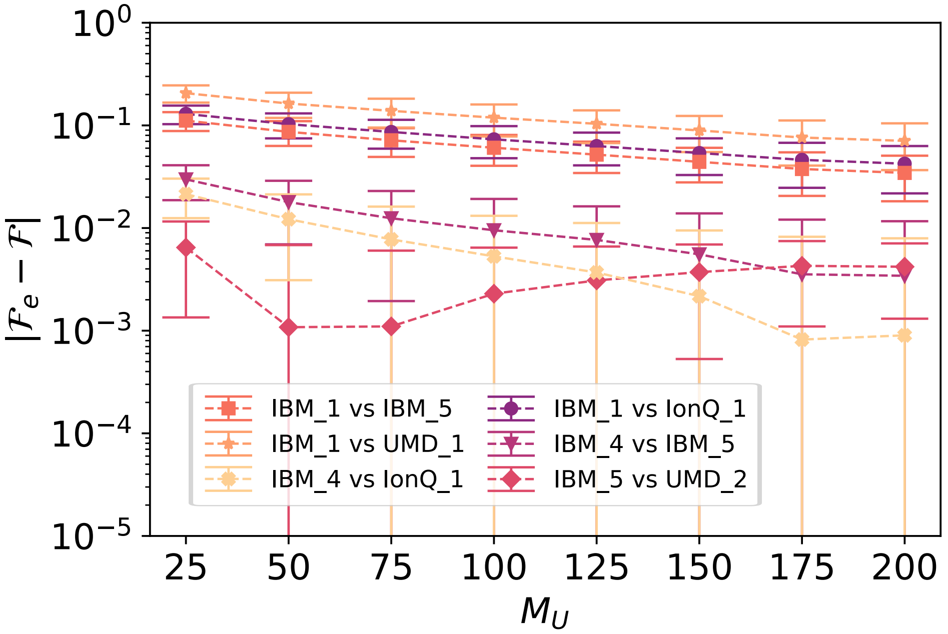


Figure S4: Fidelity error, $|\mathcal{F}_e - \mathcal{F}|$, for six randomly selected 5-qubit GHZ state cross-platform fidelities implemented on different platforms vs. the number of randomized measurements $M_U$. The number of measurement is $M_S = 2000$ for all cases.

## S3 SWAP overhead for quantum volume circuits

Two-qubit gates on non-nearest-neighbor pairs are not directly available on superconducting quantum computers. To realize such non-nearest-neighbor two-qubit gates effectively, SWAP gates are necessary. Note each SWAP gate consists of three CNOT gates. Thus, when used, non-trivial degradation to the overall fidelity of the computational output state is incurred.

Optimizing the so-called qubit routing can effectively decrease the number of involved non-nearest-neighbor two-qubit gates in evaluating the quantum volume circuits. As the number of layers $d$ increases though, the non-nearest-neighbor two-qubit gate becomes unavoidable. In Fig.

S5 we show the mean value of two-qubit gates used to implement quantum volume circuits of $d$ layers on different platforms. The value grows linearly with $d$.

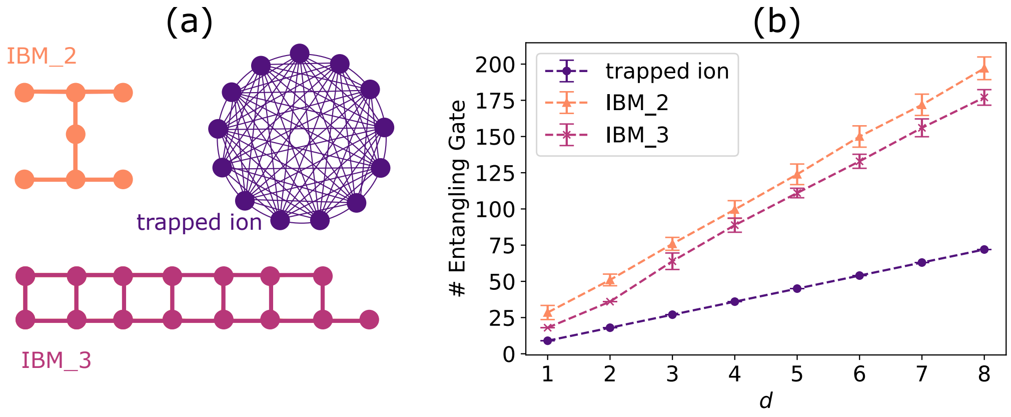


Figure S5: (a) Connectivity graphs of IBM_2, IBM_3, and trapped ion (UMD_1 as an example) (b) Average number of two-qubit (entangling) gates used to implement quantum volume circuits with $d$ layers, on different quantum computers. The trapped ion quantum computers have an all-to-all connectivity.

## S4 Quantum systems

In this section we detail the quantum systems used in this study.

### IBM Quantum Experience

We use IBM Quantum Experience service to access several of their superconducting quantum computers [5]. The ones we used are *ibmq_belem* (IBM_1), *ibmq_casablanca* (IBM_2), *ibmq_melbourne* (IBM_3), *ibmq_quito* (IBM_4), and *ibmq_rome* (IBM_5). All the IBM systems we used use superconducting transmon qubits. Their gate set is made of arbitrary single-qubit rotations on every

qubit and two-qubit CNOT gates over qubit pairs that are connected according to their respective connectivity graphs. The size of of single-qubit gate errors in the IBM systems ranges from $3.32 \times 10^{-4}$ to $5.03 \times 10^{-2}$, and the size of two-qubit gate errors ranges from $7.47 \times 10^{-3}$ to $1.07 \times 10^{-1}$. Detailed specifications of each quantum device including qubit-connectivity diagrams can be found on (https://quantum-computing.ibm.com/). We used QISKit open-source software [6] for the circuit synthesis and optimization for all of our experiments conducted on the IBM systems.

**TI_EURIQA (UMD_1)**

Error-corrected Universal Reconfigurable Ion-trap Quantum Archetype (EURIQA) is a trapped-ion quantum computer currently located at the University of Maryland. This quantum computer supports up to 13 qubits in a single chain of 15 trapped $^{171}\mathrm{Yb}^{+}$ ions in a microfabricated chip trap [7]. The system achieves native single-qubit gate fidelities of 99.96% and two-qubit XX gate fidelities of 98.5-99.3%[8]. On this platform, we compile the circuits to its native gate set through KAK decomposition. We optimize the qubit assignment through exhaustive search to minimize the anticipated noise of entangling gates. No SPAM correction was applied in post-processing.

**TI_UMD (UMD_2)**

The second trapped-ion quantum computer system at Maryland is part of the TIQC (Trapped Ion Quantum Computation) team. This quantum computer supports up to nine qubits made of a single chain of $^{171}\mathrm{Yb}^{+}$ ions trapped in a linear Paul trap with blade electrodes [9]. Typical single- and two-qubit gate fidelities are $99.5(2)\%$ and $98-99\%$, respectively. On this platform, we compile the

quantum volume to its native gate set through KAK decomposition. We apply SPAM correction to mitigate the detection noise assuming that the preparation noise is negligible.

### IonQ (IonQ_1 and IonQ_2)

The commercial trapped-ion quantum systems used by IonQ contain eleven fully connected qubits in a single chain of $^{171}\mathrm{Yb}^{+}$ ions trapped in a linear Paul trap with surface electrodes [9]. The single-qubit fidelities are $99.7\%$ for both systems at the time of measurement, while two-qubit fidelities are $95-96\%$ and $96-97\%$ for IonQ_1 and IonQ_2 respectively. On this platform, we apply the technique described in Ref. [10] to optimize the circuit. No SPAM correction was applied in post-processing.

## S5 Intra-Technology Similarity through Principal component analysis

We consider quantum states reconstructed from the shadow tomography measurement

$$\rho = \frac{1}{T}\sum_{U,b}\hat{\rho}_{U,b}, \tag{S1}$$

where $\hat{\rho} = \bigotimes_{i=1}^{n}(3U_i|b\rangle\langle b|U_i^{\dagger} - I)$ and $T$ is the number of classical shadows. After averaging over $T$ classical shadows, we decompose the density matrix defined in Eq. (S1) as a linear combination of Pauli string operators, $\rho = \sum_{k=0}^{4^n-1} v_k P_k$, where $P_k$ is the Pauli string operator and $v_k = \frac{1}{2^n}tr(\rho P_k)$. Therefore, a density matrix $\rho$ can be represented by a $4^n$-dimensional vector $\vec{v} = \sum_{k=0}^{4^n-1} v_k \hat{e}_k$. We define the vector $\vec{v}$ as the feature vector for the principal component analysis.

In the noiseless limit, the vector that represents the target state $|\psi\rangle$ is $\vec{v}_t = \sum_{k=0}^{4^n-1}\langle\psi|P_k|\psi\rangle\hat{e}_k$.

However, in the presence of noise, the vector can deviate from $\vec{v}_t$. Specifically, there are two main sources of noise: the noise from the finite sampling of the classical shadow and the noise from the imperfections in the quantum devices such as coherent and incoherent errors. The first type of noise gives a random fluctuation of the feature vector, which scales as $O(\frac{1}{\sqrt{T}})$. The second type of noise is highly platform specific. We conjecture the intra-technology similarity, i.e. quantum states prepared on similar quantum devices suffer from similar noise: The feature vectors prepared on similar devices cluster together in the $4^n$- dimensional space.

In order to observe the intra-technology similarity, we prepare the feature vectors $\vec{v}$ of all the platforms, for the 7-qubit QV states with $d = 2$ and $d = 3$. For each platform and each QV state, we independently sample $N_{\text{sample}}$ feature vectors. We define a $N_{\text{sample}} \times 4^n$ data matrix $M$. Each row of $M$ contains a feature vector $\vec{v}$. We perform the principal component analysis to project the data matrix $M$ onto a lower dimensional space in order to reduce the dimensionality of the feature vector space while preserving as much of the variation of the data as possible. The implementation of principal component analysis is based on scikit-learn. Each low-dimensional vector corresponds to a state reconstructed from the classical shadow. In the main text Figure 3(c), we can see that the low-dimensional feature vectors cluster by platform. In addition, we also perform PCA analysis for 5-qubit GHZ states. The projection result to first two principal components is shown in Fig. S6. We observe the intra-technology similarity since the data generated from similar platforms are clustered together.

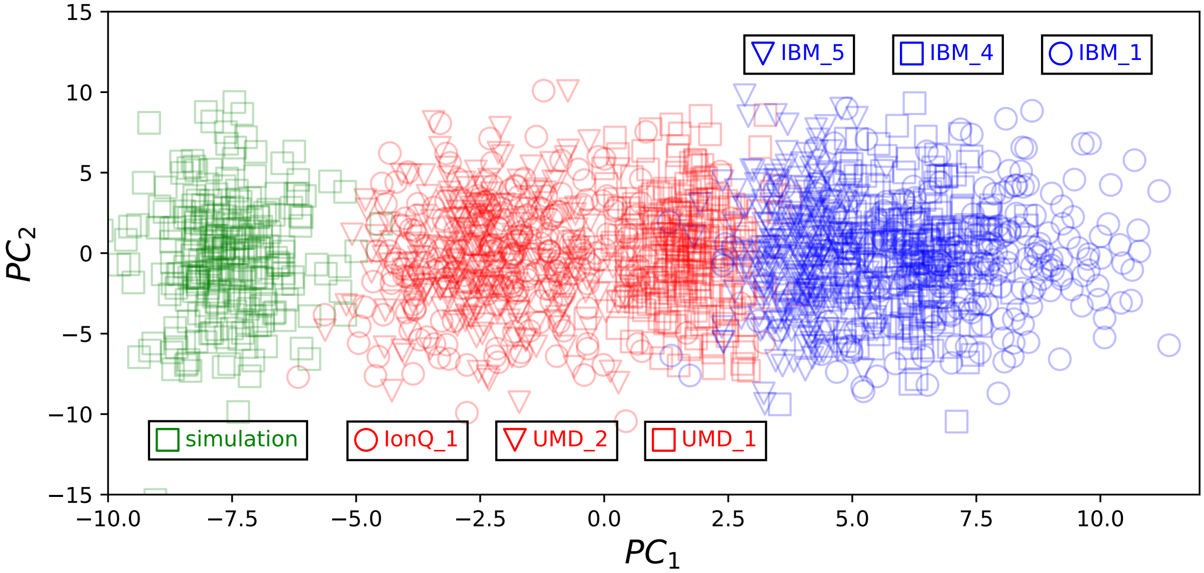


Figure S6: The projection of randomized measurement dataset of the GHZ state onto the first two principal axes, $PC_1$ and $PC_2$.

| | IBM_2 7q_2l (no calibrated) | IBM_2 7q_2l (calibrated) | IBM_2 7q_3l (no calibrated) | IBM_2 7q_3l (calibrated) |
|---|---|---|---|---|
| IBM_3 | 0.53(1) | 0.542(7) | 0.294(9) | 0.30(4) |
| UMD_1 | 0.45(1) | 0.461(8) | 0.157(6) | 0.159(5) |
| UMD_2 | 0.50(1) | 0.511(5) | N/A | N/A |
| IonQ_1 | 0.42(1) | 0.425(2) | N/A | N/A |
| IonQ_2 | N/A | N/A | 0.156(7) | 0.161(4) |
| simulation | 0.41(1) | 0.418(6) | 0.143(6) | 0.150(5) |

Table S1: The cross-platform fidelity between the platform IBM_2 and other platforms for the result with and without measurement error calibration.

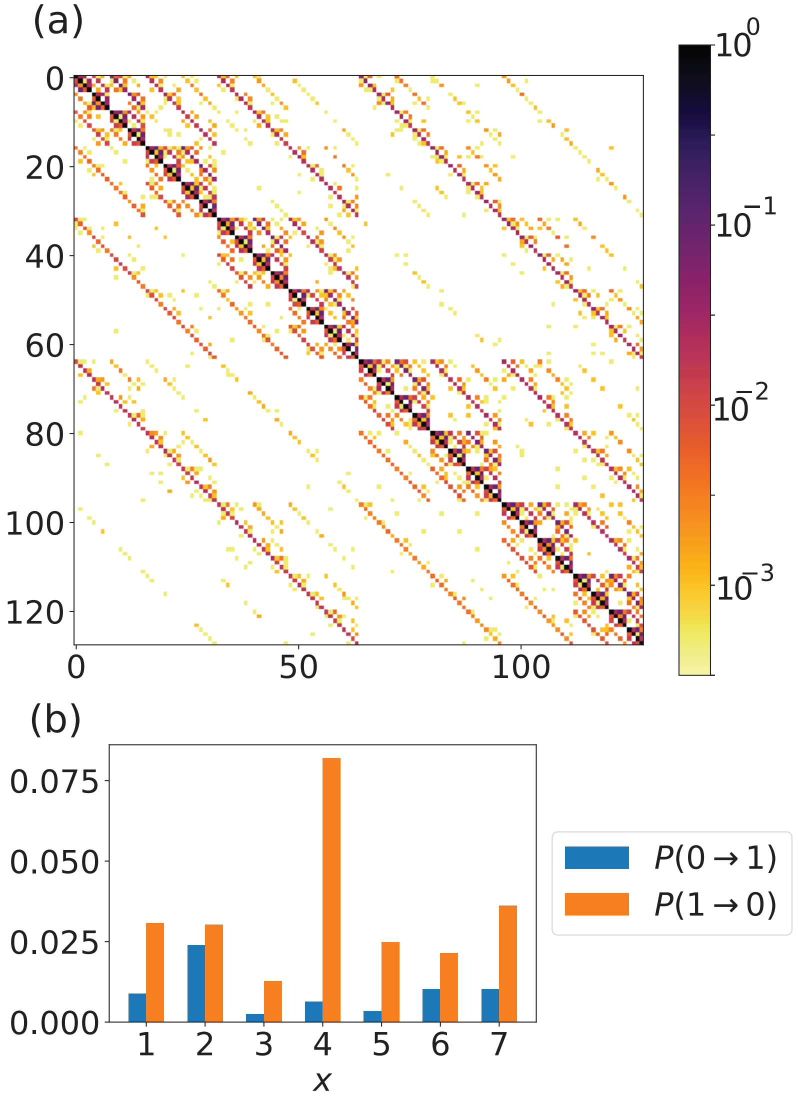


Figure S7: (a) The measurement error matrix for platform IBM_2. (b) The single-qubit measurement error for $x$th qubit.

## S6 Measurement error mitigation

In this section, we introduce the measurement error of and the error mitigation technique used for the IBM superconducting qubits. Measurement error is one of the dominant errors in the superconducting qubit devices. A measurement error manifests itself as either a $|0\rangle$ state being read as a $|1\rangle$ state or vice versa. For a quantum computer with $n$ qubits, the measurement error can be fully described by a $2^n \times 2^n$ measurement error matrix $M$. The matrix element $M[s, s']$ is the probability of measuring outcome $s'$ when the quantum computer is in state $|s\rangle$. Therefore, if we take the probability vector $P_{\text{ideal}}$ describing the ideal measurement results for a given circuit, applying the measurement matrix $M$ gives a good approximation of the results when measurement noise is present. In particular,

$$P_{\text{noisy}} = M P_{\text{ideal}}. \tag{S2}$$

In order to approximate the $P_{\text{ideal}}$, we perform an optimization to minimize the cost function

$$||P_{\text{noisy}} - M P_{\text{ideal}}||_2^2, \tag{S3}$$

subject to constraints $0 \leq P_{\text{ideal}}(s) \leq 1$ and $\sum_s P_{\text{ideal}}(s) = 1$.

For the devices IBM_2 and IBM_3 with seven qubits, we measure the measurement error matrix $M$ by initializing the qubits in all the $2^7$ possible bit strings and measure each state with 2048 shots. The measurement error matrix for IBM_2 is shown in Fig. S7(a). The dominant measurement error is the single qubit flip. In particular, the error rate for measuring $|1\rangle$ when the state is $|0\rangle$ ranges from $1\%$ to $8\%$. The error rate for measuring $|0\rangle$ if the state is $|1\rangle$ ranges from

$0.1\%$ to $2\%$, as shown in Fig. S7(b). We present the calibrated cross-platform fidelity between the platform IBM_2 and other platforms in Table S1. The calibrated result are close to the result without calibration up to the error bar.

## Supplementary References


1. Elben, A. *et al.* Cross-platform verification of intermediate scale quantum devices. *Phys. Rev. Lett.* **124**, 010504 (2020).

2. Huang, H.-Y., Kueng, R. & Preskill, J. Predicting many properties of a quantum system from very few measurements. *Nature Physics* **16**, 1050–1057 (2020).

3. Rath, A., van Bijnen, R., Elben, A., Zoller, P. & Vermersch, B. Importance sampling of randomized measurements for probing entanglement. *arXiv preprint arXiv:2102.13524* (2021).

4. Efron, B. & Gong, G. A leisurely look at the bootstrap, the jackknife, and cross-validation. *The American Statistician* **37**, 36–48 (1983).

5. Ibm quantum. https://quantum-computing.ibm.com/, 2021 .

6. Cross, A. The ibm q experience and qiskit open-source quantum computing software. In *APS March Meeting Abstracts*, vol. 2018, L58–003 (2018).

7. Maunz, P. L. W. High optical access trap 2.0. (2016).

8. Egan, L. *et al.* Fault-tolerant operation of a quantum error-correction code. *arXiv preprint arXiv:2009.11482* (2020).

9. Debnath, S. *et al.* Demonstration of a small programmable quantum computer with atomic qubits. *Nature* **536**, 63–66 (2016).

10. Nam, Y., Ross, N. J., Su, Y., Childs, A. M. & Maslov, D. Automated optimization of large quantum circuits with continuous parameters. *npj Quantum Information* **4**, 1–12 (2018).
